# *CDKN2A* Polymorphism in Melanoma Patients in Colombian Population: A Case-Control Study

**DOI:** 10.1155/2020/7458917

**Published:** 2020-10-10

**Authors:** Jose D. Tovar-Parra, Luz D. Gutiérrez-Castañeda, Sebastián R. Gil-Quiñones, Jhon A. Nova, Leonardo Pulido

**Affiliations:** Hospital Universitario-Centro Dermatológico Federico Lleras Acosta, E.S.E., DC, Bogotá, Colombia 111511, Colombia

## Abstract

**Introduction:**

Melanoma is the most aggressive type of skin cancer, with poor prognosis in advanced stages. The incidence and mortality rates have increased in recent years. Single nucleotide polymorphisms p.R24P, p.M53I, p.G101W, p.V126D, and p.A148T in the *CDKN2A* (HGNC ID: 1787) gene have been associated with the development of melanoma in different populations; however, this association has not been studied in Colombia.

**Methods:**

Cutaneous melanoma patients and healthy controls (85 cases and 166 controls) were included in this study. These subjects were screened through HRM-qPCR assay and detected variants in exon 1 and 2 of *CDKN2A* gene and confirmed with Sanger sequencing. Chi-square test was used to compare allele and genotype distributions between cases and controls. Odds ratio (OR) with 95% confidence interval (CI) was calculated to determine the association between polymorphisms and haplotypes with melanoma susceptibility. Statistical and haplotype analyses were performed using Stata® and R-Studio®.

**Results:**

Fifty-four percent of women were identified both in cases and controls. The frequencies of melanoma subtypes were 36,47% lentigo maligna, 24,71% acral lentiginous, 23,53% superficial extension, and 15,29% nodular. Variants in the *CDKN2A* gene were 11.76% in cases and 8.43% in controls. The most frequent was p.A148T in 5.88% of cases and in 4.82% of controls. GGTTG haplotype showed statistically significant differences between cases and controls (*p* value = 0.04).

**Conclusion:**

*CDKN2A* polymorphisms p.G101W, p.R24P, p.M53I, and A148T are not associated with melanoma susceptibility in the Colombian population; further studies regarding genetic interaction and additive effects between more variants are required.

## 1. Introduction

Melanoma is the most aggressive type of skin cancer and has a poor prognosis in advanced stages [1]. The incidence and mortality rate has increased in recent years, with 26% in the mortality rates as reported by Globocan [2, 3]. The etiology of melanoma is driven by the interaction between environmental factors (UV radiation) [4]; phenotypic features (red or blond hair, blue or green eyes, light skin phototypes, freckles, multiple melanocytic nevi, and the presence of nevus clinically atypical) [5]; and genotypic features such as mutations, single nucleotide polymorphisms (SNPs), and family history of melanoma [6]. SNPs have been used as markers to identify genetic susceptibility to develop any disease [7]. Currently, more than 280 genes and approximately 1,140 polymorphisms or variants related to the development of melanoma have been described in individuals of different populations [8]. The relation between the development of melanoma and the presence of certain variants depends on the population analyzed.

It has been shown that the presence of variants in certain genes leads to the development of melanoma. *CDKN2A* gene is one of the most frequent tumor suppressors genes altered between 50 and 80% of melanomas [9]; it encodes p16 and p14ARF proteins, which act as negative regulators in the transition of the G1/S and G2 phase of the cell cycle [10]. SNPs in this gene have been associated with melanoma susceptibility [7, 9–11].

Germline mutations frequencies vary across populations between 5 and 72% [12]. For the familiar melanoma *CDKN2A* gene, 22 variants have been described, 8 of these as likely pathogenic and 10 as uncertain significance [13]. Computational analysis and laboratory data coincide that the p.A148T variant is a low risk one, while variants such as p.R24P, p.M53I, p.G101W, and p.V126D are considered as pathogenic. The presence of the variants p.G101W, p.R24P, p.M53I, and p.A148T affects the function of the *CDKN2A* gene, causing the loss of interaction of p16 or p14 with client proteins such as CDK4 and CDK6. p.M53I and p.R24P variants decrease protein binding to CDK4, while the p.G101W variant decreases protein binding to CDK4 and CDK6. Also, p.M53I and p.V126D variants interaction with CDK6 is still unknown [13–16]. These variants have been associated with the development of melanoma in different populations like in Australian, Italian, Spanish, North American, English, Polish, and French [17–21].

As the aforementioned polymorphisms are associated with the risk of melanoma, the objective of this study is to identify the association between *CDKN2A* variants and the susceptibility to melanoma development in the Colombian population, by means of case-control design.

## 2. Materials and Methods

### 2.1. Study Design and Patients

An analytical, observational, case-control study was performed in Hospital Universitario Centro Dermatologico Federico Lleras Acosta E.S.E in Bogotá D.C, Colombia, during the years 2018-2019.

Eighty-five cases with a biopsy-confirmed diagnosis of cutaneous melanoma and 170 healthy controls (matched 1 : 2 by age, sex, and Fitzpatrick Skin Phototypes) with no prior personal or familial history of melanoma were identified. Four control samples were excluded due to poor quality. Potential controls with any suspicious pigmented skin lesion were not included. The study protocol was approved by the Research Ethics Committee of the University Hospital-CDFLLA and follows international research standards of the declaration of Helsinki. Each patient signed an informed consent form for the use of their clinical information and molecular analysis from blood samples.

### 2.2. DNA Preparation and Mutation Screening

Genomic DNA was extracted from total blood using a QIAamp® DNA Mini, and Blood Mini Handbook kit (cat. #69506 Qiagen, Hilden, Germany) was used according to the manufacturer's protocol. DNA was eluted in nuclease-free water (cat. #7732-18-5 Amresco); then, it was quantified by the use of NanoDrop® (Thermo Fisher Scientific). To detect variants, we amplified exon 1 and 2 of the *CDKN2A* gene by PCR in at least 2 separate preparations of genomic DNA. The primers sequences were used as shown in Table 1.

PCR conditions were as follows: initial denaturation for 3 min at 95°C; 40 cycles of denaturation for 30 sec at 95°C, hybridization for 30 sec (between 53°C and 65°C depending on the exon), and 30 sec extension at 72°C; and a final extension for 3 min at 72°C. The PCR products were purified using a QIAquick PCR purification kit (cat. #28104 Qiagen®). Sequencing was performed with the Sanger method using a BigDye Terminator V1.1 Cycle Sequencing Kit and an ABI PRISM 3130xl Genetic Analyzer (Applied Biosystems ®). Sequence analysis was performed using Chromas (free version) and NovoSNP version 3.0 (free version from the internet, http://www.molgen.ua.ac.be/bioinfo/novosnp/index.html) software. All sequences were confirmed by a second reviewer.

### 2.3. Melting Curve Acquisition and Analysis

All *CDKN2A* primers are listed in Table 1. Calibration of CFX96 Touch tm Real-Time PCR (BIO-RAD) equipment was done with Melt Calibration Kit (cat. # 184-5020 BIO-RAD) according to the manufacturer's protocol. The samples were processed with Precision Melt Supermix (cat. #172-5112 BIO-RAD). Each reaction mix contained 4 *μ*l DNA (10 ng/*μ*l), hot-start iTaqTM DNA polymerase, dNTPs, MgCl2, and EvaGreen. The Real-Time PCR (qPCR) conditions were as follows: initial denaturation for 2 min at 95°C; 45 cycles of denaturation for 10 sec at 95°C, annealing for 30 sec (between 53°C and 65°C depending on the exon), and 30 sec extension at 72°C. The High-Resolution Melting Analysis (HRM) was immediately performed from heteroduplex formation 30 sec at 95°C, 1 min at 60°C, and High-Resolution Melting cycle 10 sec/step at 65-95°C with 0.2°C increments.

**Figure 1:**
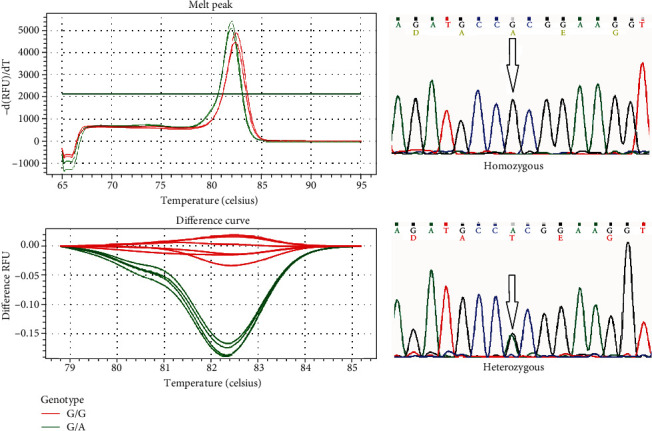
Exon 2 of *CDKN2A* gene. Left side: HRM curves with the mutations c.442G>A-p.A148T (rs3731249) in normalized and temp-shift melting curve and difference plot. Right side: chromatograms for negative control (WT DNA) and positive case (heterozygous).

The differences between normalized melting curves of cases and controls and wild-type sequence control curves were determined by comparison using the Precision Melt Analysis™ v1.1 Software Upgrade, and samples were assigned to different groups according to the positive and negative controls. DNA samples from 10 patients were sequenced randomly for each amplificated in order to be used as a control in HRM-qPCR genotyping. From the sequencing results, the samples presenting the variants (p.R24P, p.M53I, p.G101W, p.V126D, and p.A148T) were taken as positive controls, the rest of the samples as negative controls (Figure 1).

### 2.4. Statistical Analysis

Statistical analysis was performed using a licensed version of Stata 16®. The normal distribution of variables was determined for both groups using Shapiro-Wilk test. Genotyping results were compiled, and genotype, allele, and haplotype frequencies were calculated manually in Microsoft Excel®. Hardy Weinberg Equilibrium for the control group was determined by Chi-square test (*χ*^2^). Genotype and Allele distribution of p.R24P, p.M53I, p.G101W, p.V126D, and p.A148T were compared using a *χ*^2^ test. Odds ratio (OR) with a 95% confidence interval (CI) was used to determine genetic association. Statistical significance was defined as *p* value < 0.05. R-Studio was used to confirm Haplotypes, with the Haplo.Stats package version 1.7.7 (Haplotype Score Tests or Regression Models).

## 3. Results

Eighty-five cases and 166 controls were included; the mean age was 59, 41 ± 15.6 years for cases and 57, 91 ± 14.92 for controls. Women accounted for 54,12% in cases and 53,61% in controls. 81.18% of cases and 91.57% of controls were born in the central region of Colombia (Cundinamarca).

According to the phenotypic and clinical characteristics, both cases and controls suit in the phototypes 2 (19%), 3 (70%), and 4 (11%). There were fair-haired and fair-eyed patients in the group of cases, but the dark features were the most prevalent in both groups (Table 2). History of familial melanoma was seen in 8,24% of cases and 1,2% in controls (1,2%). In terms of the number of nevus, no control displayed more than 100; however, 3,52% was reported in cases. On the other hand, controls displayed a count of 50-100 nevus in a 3,01% proportion, compared with a 12,94% in cases.

In the cases (*n* = 85), melanoma subtypes were displayed as following: 36,47% (*n* = 31) lentigo maligna melanoma (LMM), 23,53% (*n* = 20) superficial spreading melanoma (SSM), 24,71% (*n* = 21) acral lentiginous melanoma (ALM), and 15,29% (*n* = 13) nodular melanoma (NM). Pathologic studies revealed a Clark level of IV in 41,18% of the cases and a Breslow thickness >1 in 24,71%. Mitoses were present in 51,76% and ulceration in 21,18% of the patients (Table 2).

### 3.1. *CDKN2A* Variant Analysis

Frequencies of *CDKN2A* variants found between cases and controls were 11.76% and 8.43%, respectively. Frequencies of variants are shown as following: p.R24P (3,52% vs. 2,40%), p.M53I (0% vs. 0%), p.G101W (2,35% vs. 0%), p.V126D (2,35% vs. 4,82%) and p.A148T (5,88% vs. 4,82%) in cases and controls, respectively. According to the genotype, all variants were heterozygous, and there was no association between the analyzed variants and the risk of developing the melanoma (Table 3).

Overall, 26 variants were identified in total, 10 cases had 12 variants distributed as follows: 3 variants were p.R24P, 2 were p.G101W, 2 were p.V126D, and 5 were p.A148T. Two cases present 2 variants simultaneously. According to the subtype melanoma, 4/10 were SSM, 3/10 ALM, 2/10 LMM, and 1/10 NM. According to the phototypes, 1/10 were phototype 2, 7/10 phototype 3, and 2/10 phototype 4. Ten percent of the cases had European ancestry (most frequently German Ancestry), and 80% had a familiar history of cancer. While 14 controls had 14 variants distributed as follows: 4 variants were p.R24P, 2 p.V126D, and 8 p.A148T.

### 3.2. *CDKN2A* Haplotype Frequencies

We estimated the haplotype frequencies in melanoma patients and healthy controls (Table 4). This analysis shows that haplotypes (1 and 4) were the most common in both the melanoma patients haplotype 1 freq: 0.94, and haplotype 4 freq: 0.023 and the healthy controls haplotype 1 freq: 0.95 and haplotype 4 freq 0.024. According to this result, it is shown that both groups have similar frequencies. However, we confirmed that there are differences between cases and controls in three haplotypes (5, 6, and 7). The frequencies were as follows: haplotype 5 = 0.00584, haplotype 6 = 0.00573, and haplotype 7= 0.01176 (Table 4).

Finally, we found that the haplotype 7 (GGTTG) had significant differences between case and control groups (*p* value = 0.04). Also, we found similar *p* values when we estimated the association between sex and the presence of this haplotype (*p* value = 0,04).

## 4. Discussion

The incidence of cutaneous melanoma (CM) has increased in recent decades [22]. However, it varies according to ethnic group and geographic location, with a clear predilection in countries with a predominantly light-skinned population such as the United States, which report an incidence of 19,7/100.000 inhabitants according to the “SEER” database of the National Cancer Institute [23]. Other populations such as European report incidences of 9,9-14,6/100.000 cases per year [22, 23]. Less frequently, non-Caucasian populations such as South America, Central America, The Pacific, Africa, and Asia show incidences of less than 4/100.000 inhabitants per year [2, 23]. However, the incidence in the Colombian population has increased in recent years. For this reason, it is important to know about the variant that generates susceptibility or risk for the development of melanoma in the Colombian population.

In our study, we analyzed 85 melanoma patients and 166 healthy control. Controls were chosen based on the paring of age, sex, and phototype with cases. Regarding the age, the average in the case and control groups was 59.41 and 57.91, respectively. These data are in accordance with the presentation age of disease in other populations, which are found between 55 and 65 years [24]. The analyzed population in this research resides in a mountainous Colombian region (Andean Mountain areas), which is located over 2600 m above sea level and where UV radiation indexes are higher than 10 throughout the year. Solar exposition is one of the risk factors related to the development of specific subtypes of melanoma [25]. However, melanoma incidence is lower than in other regions of the world [2].

Like many other countries in Latin America, Colombia is marked by ancestral diversity as a result of the mixture [26] of three major ethnic groups: Amerindians (descendants of indigenous people), European immigrants (mostly Spanish), and Africans [27]. This may be one of the reasons behind the dissimilar epidemiological pattern shown in our study, in which LMM and ALM were the most common cutaneous melanoma subtypes, comprising 36,46% and 24,71%, respectively. These data are in concordance with data of a different patient cohort previously studied in the same geographical region and with data reported in a different geographical region of Colombia (1285 m above sea level) [28]. Contrary to what was reported by Pozzobon et al., in which predominate SSM (42,7%), LMM (33,7%), and ALM (18.3%) subtypes [29]. On the other hand, worldwide the most frequencies subtypes of melanoma are SSM between 50 and 70% followed by NM [29, 30]. Although the presentation pattern in our study was different from the one reported worldwide, within the clinical-pathological characteristics of our study, 47.06% presented a Clark level ≥ III and 38.83% a Breslow index ≥2.0 mm, 21.18% had ulcer lesion, and 43,53% tumor was located on the head and neck.

This is the first study in the Colombian population that sought to determine the frequencies of the polymorphic variants of the *CDKN2A* gene and their implication in the susceptibility of melanoma. Some studies have reported frequencies of some variants in this gene, such as Brazilian, Uruguayan, and other populations [18, 19, 31, 32]. Approximately 20-40% of melanomas occur in patients with a family history of melanoma [12, 33], and 45% of these are the due inheritance of a mutation or a predisposition gene [34]. Within familial melanoma syndromes, germline mutations of *CKDN2A* and *CDK4* genes remain the most common [34].

In this study, we found that 8.42% had a family history of melanoma and 7,05% of melanoma patients had recurrence. Ten percent out of patients with variants had present European ancestry. These data are similar as the one reported by Borges et al. in the Uruguay population, who described the presence of the p.G101W variant in 3/6 Uruguayan families, which were associated with familial melanoma, and these families presented ancestors of southern Europe (Italy) [31]. On the other hand, a study carried out with 127 patients and 128 controls in the south of Brazil found the relation between the presence of the p.A148T variant and the development of melanoma (12,6% vs. 3,9% controls). Patients with the variant reported European ancestry (Germany) [32].

In this study, we genotyped 5 *CDKN2A* variants which have been identified as pathological: p.R24P, p.M53I, p.G101W, p.V126D, and p.A148T. Ten cases and 14 controls presented these variants, and 2 cases presented two variants simultaneously. Ten percent of the cases presented European ancestry (Germany), concordant with other variants in the *CDKN2A* gene in other Latin American populations such as Uruguay (p.G101W) [31], Brazil (p.A148T) [32], and Chile (p. M53I) [12]. Otherwise, we did not find associations between variants and the development of melanoma. The haplotype analysis was performed including all variants; our analysis yielded a statistically significant value for GGTTG haplotype segregation (**p** = 0.047). However, this haplotype has not been previously analyzed in another population.

In both cases and controls, we detected the p.R24P variant, which was previously identified in the Australian and British population [19, 35]. We did not identify the p.M53I variant, but it has been reported in the Chilean population [12]. Contrary to this, we detected the p.G101W variant in two cases, which has been previously reported in the Austrian [36], Uruguayan [31], Ligurian [37], French [19], Australian, Spanish, and British population [21, 38]. p.V126D variant has been previously detected in North American patients with familiar history of melanoma; however, in this study, the variant was detected in patients and healthy controls without familiar history of melanoma [39]. Similarly, the p.A148T variant was detected in patients and controls; this variant has been reported in the different population as Brazilian [40], Australian [18], Israeli [41], and Greek [5].

Alanine by threonine, substitution (p.A148T) has been considered one of the most common variants in cutaneous melanoma and has been identified in familial melanoma cases. A study performed in Polish population with 471 cases, and 1271 controls identified an increased risk (OR 3.5) for patients diagnosed under 50 years; thus, this variant has been shown to increase susceptibility to melanoma [42]. Although, different reports indicate that the p.A148T variant does not alter protein function; other studies show that the p.A148T is in strong linkage disequilibrium with a change in the promoter region at the c.-493 position, which reduces its expression and affects the p16 protein function [1, 42]. p.A148T significance is controversial, since several studies have not shown evidence in its role as a melanoma susceptibility allele, but others have [43–45].

The p.V126D variant was detected both in cases and controls, this variant has been found in populations as Italian, French, North American, and Swiss as a high-risk variant for melanoma [46]. The presence of this variant among cases and controls in our population may be due to admixture between Native Americans, European, and Africans [47]. The p.G101W variant was detected only in cases but not in controls, which correlates with previously reported populations from Argentina, Uruguay, Brazil, Spain, Italy, and France at different frequencies [12]. These data are like to findings in this study regarding the p.R24P variant. According to reports in other studies with Austrian population, this variant has been considered as a pathogenic variant such as p.G101W and p.V126D, conversely p.A148T as benign variant [36]. We found that the prevalence of pathogenic variants in *CDKN2A* was 5,88% for cases without a family history of CM, but other studies have reported a prevalence of approximately 1% [21].

Our results showed the frequencies of the *CDKN2A* variants in the Colombian population are consistent with data reported in other populations. As the possibility of biological bias exists in the present study, it is likely that our differences are merely ethnical, environmental, or due to modifying genetic variants that may interact with the polymorphisms studied. Further studies regarding Latin American populations are indispensable in order to establish more precise associations between genetic polymorphisms and susceptibility to melanoma.

## 5. Conclusion

In conclusion, the variants analyzed in this study allowed us to elucidate the frequencies between both melanoma patients and healthy people. p.G101W variant was only displayed in melanoma patients, and the haplotype analysis revealed the significance of this variant in genetic segregation. Additionally, the most frequent variant in cases and controls was the p.A148T variant with 5.88% and 4.82%, respectively. Variant p.M53I was not found neither in cases nor in controls. To avoid biases related to this type of analysis, matching by sex, age, and phototype was performed. This allowed us to generate knowledge at a national level of the behaviour of the variants in the Colombian population.

## Figures and Tables

**(a):** 

Conventional PCR				
Exon	Primers 5′-3′	Product size (pb)	Melting temp. (°C)	Nucleotide sequence 5′-3′
1	F	399	61.0	CTGCAAACTTCGTCCTCCAGA
	R			CCCTTGCCTGGAAAGATACC
2	F	479	61.0	GGTGAGGGGGCTCTACACAA
	R			GGTCTCCCGGGCTGAACTTT

**(b):** 

HRM–qPCR assay						
RS	SNP	Protein change	Primers 5′-3′	Product size (pb)	Melting temp. (°C)	Nucleotide sequence 5′-3′
rs104894097	c.71G>C	p.R24P	F	79	63.3	CCGCCTCCAGCAGCG
			R			ATGGAGCCTTCGGCTGACT
rs104894095	c.159G>C	p.M53I	F	121	59.0	GGGCTCTACACAAGCTTCCTT
			R			GTGGAGCAGCAGCAGCT
rs104894094	c.301G>T	p.G101W	F	99	61.0	GAGGGCTTCCTGGACACG
			R			CTCCTCAGCCAGGTCCAC
rs104894098	c.377T>A	p.V126D	F	88	61.0	CGTGGACCTGGCTGAGGA
			R			GGCATGGTTACTGCCTCTGG
rs3731249	c.442G>A	p.A148T	F	100	61.0	GGGCACCAGAGGCAGTAAC
			R			GCTTTGGAAGCTCTCAGGGTA

**Table 2:** The demographic and clinical characteristics of melanoma cases and controls.

Feature	Cases		Controls	
	*n* = 85		*n* = 166	
	*N*	%	*N*	%
Age				
Mean	59,41 (24-92)	±15.6	57,91 (20-88)	±14.92
Sex				
Women	46	54,12%	89	53,61%
Men	39	45,88%	77	46,39%
Melanoma subtype				
Superficial spread	20	23,53%	—	—
Nodular	13	15,29%	—	—
Acral lentiginous	21	24,71%	—	—
Lentigo maligna	31	36,47%	—	—
Variant				
p.R24P				
Presence	3	3,52%	4	2,40%
Absence	82	93,78%	162	97,60%
p.M53i				
Presence	0	0	0	0
Absence	85	100%	166	100%
p.G101W				
Presence	2	2,35%	0	0
Absence	83	97,65%	166	100%
p.V126D				
Presence	2	2,35%	2	1,20%
Absence	83	97,65%	164	98,80%
p.A148T				
Presence	5	5,88%	8	4,82%
Absence	80	94,12%	158	95,18%
Clark level				
Negative	33	38,82%	—	—
I	2	2,355	—	—
II	8	9,41%	—	—
III	2	2,35%	—	—
IV	35	41,18%	—	—
V	5	5,88%	—	—
Breslow scale				
Nonreported	32	37,65%	—	—
≤1,0 mm	11	12,94%	—	—
>1,0-2,0 mm	9	10,59%	—	—
>2,0-4,0 mm	12	14,12%	—	—
>4,0 mm	21	24,71%	—	—
Mitoses				
Nonreported	32	37,65%	—	—
Negative	9	10,59%	—	—
≤1,0 mm2	17	20%	—	—
>1,0 mm2	27	31,76%	—	—
Ulceration				
Nonreported	32	37,65%	—	—
Presence	18	21,18%	—	—
Absence	35	41,18%	—	—
Location				
Trunk	12	14,12%	—	—
Head and neck	37	43,53%	—	—
Upper extremities	5	5,88%	—	—
Lower extremities	9	10,59%	—	—
Hands and feet	21	24,70%	—	—
Phototype				
2	16	18,82%	31	18,79%
3	59	69,41%	117	70,91%
4	10	11,76%	17	10,30%
Eye color				
Black or dark brown	44	51,76%	123	74,10%
Light brown	30	35,29%	27	16,27%
Green	9	10,59%	9	5,42%
Blue	2	2,35%	7	4,22%
Hair color				
Black or dark brown	55	64,71%	136	81,93%
Light brown	29	34,12	28	16,87%
Red or blond	1	1,18%	2	1,20%
Personal history of cancer				
Yes	3	3,52%	9	5,42%
No	82	96,48%	157	94,58%
Family history of cancer				
Yes	48	56,47%	78	46,99%
No	37	43,53%	88	53,01%
Personal history of melanoma				
Si	6	7,05%	0	—
No	79	92,95%	166	100%
Family history of melanoma				
Si	7	8,24%	2	1,20%
No	78	91,76%	164	98,80%
Nevus				
≤50	71	83,53%	161	96,99%
50-100	11	12,94%	5	3,01%
>100	3	3,53%	0	0%

**Table 3:** Genotype and allele distributions of variants *CDKN2A* gene in cases and controls groups.

Genotype/allele	Cases *n* = 85 (%)	Control *n* = 166 (%)	*χ* ^2^	*p* value	OR (95% CI)
p.R24P					
G/G	82 (96.47%)	162 (97.59%)	0.260	0.610	1.48 (0.32-6.77)
G/C	3 (3.53%)	4 (2.41%)			
G	167	328	0.256	0.613	1.47 (0.32-6.65)
C	3	4			
p.M53I					
G/G	85 (100%)	166 (100%)	0.115	0.735	1,94 (0.03-99.00)
G/C	0	0			
G	170	332	0.115	0.735	1.94 (0.03-98.57)
C	0	0			
p.G101W					
G/G	83 (97.65%)	166 (100%)	3.294	0.070	9.97 (0.47-210.05)
G/T	2 (2.35%)	0			
G	168	332	3.280	0.070	9.85 (0.47-206.38)
T	2	0			
p.V126D					
T/T	83 (97.65%)	164 (98.80%)	0.473	0.492	1.97 (0.27-14.27)
T/A	2 (2.35%)	2 (1.20%)			
A	2	2	0.469	0.494	1.96 (0.27-14.06)
T	168	330			
p.A148T					
G/G	80 (94.12%)	158 (95.18%)	0.129	0.719	1.23 (0.39-3.89)
G/A	5 (5.88%)	8 (4.82%)			
G	165	324	0.126	0.723	1.22 (0.39-3.81)
A	5	8			

**Table 4:** Haplotype effect model: additive.

Global score statistics									
Global − stat = 8.91587, df = 6, *p* value = 0.17836									
Haplotype-specific scores									
#	R24P	M53I	G101W	V126D	A148T	Hap-Freq	Hap-Freq case	Hap-Freq control	*p* value
1	G/G	G/G	G/G	T/T	G/G	0.95186	0.94099	0.95783	0.37088
2	G/C	G/G	G/G	T/T	G/G	0.01029	0.00607	0.01205	0.57164
3	G/G	G/G	G/G	T/A	G/G	0.00604	0.00592	0.00602	0.99435
4	G/G	G/G	G/G	T/T	G/A	0.02417	0.02368	0.02410	0.98849
5	G/C	G/G	G/G	T/A	G/G	0.00193	0.00584	NA	0.15458
6	G/C	G/G	G/G	T/T	G/A	0.00173	0.00573	NA	0.13008
7	G/G	G/G	G/T	T/T	G/G	0.00398	0.01176	NA	0.04723∗

## Data Availability

The files related to informed consent, clinical data, and molecular biology results; data used to support the findings of this study are restricted by the ethics committee of the Hospital Universitario - CENTRO DERMATOLÓGICO FEDERICO LLERAS ACOSTA. E.S.E., DC, Bogota, Colombia. However, the data are available from the file of code 1DSI02-6AE for researchers who meet the criteria for access to confidential data. These files can be requested at the following email from the ethics committee: Comitedeeticaeninvestigacion@dermatologia.gov.co.
